# Immunopathogenesis of Acute Flare of Chronic Hepatitis B: With Emphasis on the Role of Cytokines and Chemokines

**DOI:** 10.3390/ijms23031407

**Published:** 2022-01-26

**Authors:** Chieh Liu, Yi-Fen Shih, Chun-Jen Liu

**Affiliations:** 1 Department of Medicine, Chung-Shan Medical University, Taichung 40201, Taiwan; liuliujade@gmail.com; 2 Department of Physical Therapy and Assistive Technology, National Yang Ming Chiao Tung University, Taipei 11221, Taiwan; yfshih@nycu.edu.tw; 3 Hepatitis Research Center, National Taiwan University College of Medicine and Hospital, Taipei 10002, Taiwan; 4 Graduate Institute of Clinical Medicine, National Taiwan University College of Medicine and Hospital, Taipei 10002, Taiwan; 5 Department of Internal Medicine, National Taiwan University College of Medicine and Hospital, Taipei 10002, Taiwan

**Keywords:** hepatitis B, reactivation, acute flare, acute exacerbation, immunopathogenesis, cytokine, chemokine

## Abstract

Acute flares (AFs) of chronic hepatitis B usually occur during the immune-active stage (both immune clearance phase and immune reactivation phase), as the host immune system tries to control the virus. Successful host immune control over viral replication is usually presented as hepatitis B surface antigen seroclearance; however, 20–30% individuals with chronic hepatitis B may encounter repeated AFs with accumulative liver injuries, finally leading to the development of cirrhosis and hepatocellular carcinoma. AF can also develop in other clinical situations such as organ transplantation, cancer chemotherapy, and under treatment for chronic hepatitis B or treatment for chronic hepatitis C in patients with co-infected hepatitis B/hepatitis C. Understanding the natural history and immunopathogenesis of AF would help develop effective strategies to eradicate the virus and improve the clinical outcomes of patients with chronic hepatitis B. In this review article, the immunopathogenesis of AF, and the involvement of innate and adaptive immune responses on the development of hepatitis B flare will be briefly reviewed, with the emphasis on the role of cytokines and chemokines.

## 1. Introduction

When acquired early in life, the course of the chronic hepatitis B (CHB) can be separated into four stages, namely immune tolerance phase (indicated by hepatitis B e antigen [HBeAg] positivity, high serum hepatitis B virus (HBV) DNA levels, normal serum alanine aminotransferase [ALT] levels, and normal or minimal change of liver histology), immune clearance phase (positive for HBeAg, intermittent elevation of serum ALT levels, and intermittent elevation of serum HBV DNA levels), immune control phase (negative for HBeAg, serum HBV DNA levels <4 log_10_/mL, and normal serum ALT levels) and reactivation phase (negative for HBeAg, serum HBV DNA levels ≥4 log_10_/mL, and elevation of serum ALT levels) [1,2,3]. Acute flares (AFs) or acute exacerbations (AEs) of chronic hepatitis B associated liver damage usually occur during the immune-active stage (both immune clearance phase and immune reactivation phase), as the host immune system tries to control the virus and kill virus-infected hepatocytes [2,3]. Sustained host immune control over viral replication in the immune control phase can lead to hepatitis B surface antigen (HBsAg) seroclearance; however, 20–30% individuals among the CHB patients may encounter intermittent and repeated AFs with accumulative liver injuries, finally leading to the development of cirrhosis and hepatocellular carcinoma (HCC) [1,2,3,4,5,6,7,8,9,10,11,12,13,14,15,16,17]. AF can also develop in various clinical situations such as organ transplantation, cancer chemotherapy, treatment for chronic hepatitis B, and treatment for chronic hepatitis C by using direct acting antiviral (DAA) in HBV/hepatitis C virus (HCV) co-infected patients [3,15,18,19,20,21,22,23,24,25,26,27,28,29,30,31,32,33,34,35,36,37,38,39,40,41,42,43,44,45].

AF can be followed by immune control over viral replication, or contributes to the development of adverse clinical outcomes. In some cases, liver failure and even liver-related death may occur [2,3,12]. Understanding the natural history and immunopathogenesis of AF may help develop effective strategies to eradicate the virus and improve the clinical outcomes of patients with chronic hepatitis B.

In this article, the immunopathogenesis of AF of chronic HBV infection, and the involvement of innate and adaptive immune responses on the development of hepatitis B flare will be briefly reviewed, with the emphasis on the role of cytokines and chemokines.

## 2. Acute Flare of Chronic Hepatitis B

### 2.1. Definition and Clinical Impact

Chronic HBV infection can be described as a status characterized by dynamic interactions among HBV, hepatocytes and immune cells of the host [16,17]. During its natural course, AFs of hepatitis B, presented as a sharp increase of serum ALT to ≥5x upper limit of normal (ULN) or ≥3-fold increase of the baseline level, whichever is higher [5], may occur spontaneously [1]. In addition, patients under the state of immunosuppression due to cancer chemotherapy or the usage of immunosuppressive agents might be susceptible of hepatitis B flare [2]. Other conditions which could lead to AF of hepatitis activity include patients withdrawing from nucleos(t)ide analogue (NUC) therapy for chronic hepatitis B, HBV/HCV co-infected patients receiving DAAs for chronic HCV infection, or cancer patients receiving immune checkpoint inhibitors [3,15,19,20,21,22,23,24,25,26,27,28,29,30,31,32,33,34,35,36,37,38,39,40,41,42,43,44,45]. AF of hepatitis B can lead to liver failure without any prophylaxis, and thus requires clinical attention.

### 2.2. Clinical Settings

Spontaneous AF of Hepatitis B

AFs of hepatitis B usually develop in two phases during the course of chronic HBV infection: the HBeAg-positive immune clearance phase [1,4,5,8], and the HBeAg-negative reactivation phase [9,10,11]. Previous hospital-based cohort studies revealed that annual incidence of hepatitis B flare reached 27% among 358 HBeAg-positive patients and was 10% among 279 HBeAg-negative patients [4,8,9,11]. These episodes can occur repeatedly during follow-up [4,8,9,11].

The clinical features of hepatitis B flare vary greatly, from asymptomatic, acute hepatitis-like flare, to hepatic decompensation or even hepatic failure. Before the rise of serum ALT level, an increase of serum HBV DNA (HBV reactivation, HBVr) and HBsAg levels usually occur first. Recent data suggested that if the increased serum HBV DNA maintained at a high level throughout the course of AF, then the host immune responses aiming to clearing the virus might be insufficient [6,7]. Under this circumstance, active hepatocytolysis followed by hepatic decompensation may occur [12,13]. The risk of hepatic decompensation has been reported as 2.4% during HBeAg sereoclearance in previous cohort studies [4,8,9,11]. Because AFs developed in viremic cirrhotic patients are associated with a higher risk of decompensation/mortality [14], immediate antiviral therapy is needed for this subgroup of patients. For those patients who are non-cirrhotic and there is no concern of developing decompensation, a period of observation between 3 to 6 months is recommended before the decision of antiviral therapy. Because severe and repeated flares are associated with the risk of developing cirrhosis [2,3], antiviral therapy should also be considered for people experiencing repeated hepatitis flares in order to avoid progression into adverse clinical outcomes.

## 3. Cancer Chemotherapy or Immunosuppressive Therapies for Rheumatoid Diseases

Hepatitis B flares in patients who receive immunosuppressants or cancer chemotherapy have become a significant clinical concern these days [15]. Being an immune-mediated disease entity, the clinical course of hepatitis B flare is often influenced by immunosuppression followed by immune restoration in patients with chronic HBV infection who undergo immunosuppressants or chemotherapy. It has been reported in early 1990s that reactivation of HBV replication with the development of jaundice was found in HBsAg carriers with lymphoma (22%) or in patients with resolved HBV infection (2%) after the start of chemotherapy for lymphoma [5]. Liver failure was found in 7% of HBsAg-positive patients and in 2% of HBsAg-negative patients, respectively [28]. It is now universally recognized that patients with chronic HBV infection or with occult HBV infection who undergo intense immunosuppression or chemotherapy for hematologic or other solid tumors, or stem cell or solid organ transplantation are susceptible to HBV reactivation [27,28,29,30]. Clinically, HBVr is presented as an increase in serum HBV DNA during immunosuppressive therapy or cancer chemotherapy. Serum ALT elevation (hepatitis flare) may occur in-between therapy administrations or after the end of the therapy [28]. Hepatitis B flares have also been reported in 30% of the patients following trans-arterial chemoembolization of HCC [29]. Hepatitis B flare in these patients can be severe and sometimes lead to mortality, and screening and monitoring of HBV infection, and prophylactic antiviral therapy are thus mandatory for this group of patients.

Chemotherapy or immunosuppressive regimens may influence the risk of HBVr and hepatitis flare [15]. Adding corticosteroid as a part of the treatment regimen was found to increase the risk and severity of HBVr [31]. The use of B cell depleting monoclonal antibodies against CD20 (anti-CD20) such as rituximab was documented to significantly raise the risk of HBVr and hepatitis flare [34]; the negative impact of rituximab was also observed in HBsAg-negative antibody against hepatitis B core antigen (anti-HBc)-positive patients, including those who were seropositive for anti-HBs [32,33]. As shown in a recent prospective study in 150 HBsAg-negative anti-HBc-positive patients undergoing cyclic rituximab-containing chemotherapy for lymphoma, the incidence of HBVr was 10% and the incidence of hepatitis flare was 6% [33].

After the launch of oral anti-HBV agents potent in the suppression of HBV replication in 2000s, prevention of HBVr by potent anti-HBV agent is now clinically feasible and has become the standard of care in this clinical condition [15,27]. A systemic review of 14 studies revealed that the pooled incidence of HBV-related hepatitis, liver failure, and mortality decreased from 33.4% to 4%, from 13% to 0%, and from 6.7% to 2%, respectively by using prophylactic NUC (such as lamivudine) therapy [30].

## 4. Withdrawal Hepatitis Flares Post-NUC Therapy for Chronic Hepatitis B

Hepatitis B flares may develop after the cessation of antiviral NUC therapy in patients with CHB. The chronological profiles of HBV DNA versus ALT level are similar to those occurring during the spontaneous acute flare of chronic hepatitis B [17]. An increase of serum HBV DNA occurs after cessation of NUC therapy, followed by hepatitis flares [3,19]. The off-therapy hepatitis flares also resemble the spontaneous hepatitis flares in the spectrum of the clinical presentation. Hepatic decompensation and fatality may occur if the flare is undiagnosed, left retreated or not treated in time [19,20,21,22]. Although this type of hepatitis flares tends to develop after cessation of NUC therapy in the majority of the patients who remain HBeAg positive at the time point of stopping NUC [22], it may still occur in patients with NUC-induced HBeAg seroconversion, particularly in those patients with inadequate consolidation therapy post-HBeAg seroconversion [23,24,25]. Numerically, about 40~70% of patients experience clinical relapse (HBV DNA ≥ 2000 IU/mL with ALT ≥ 2X ULN) within one year after stopping NUC therapy [21,26]. Severe hepatitis activity with the risk of hepatic decompensation may occur when the clinical relapse is too late to be diagnosed and NUC therapy is not resumed in time [21].

The findings of a recent study consistently suggested that end-of-treatment serum quantitative HBsAg titer may help prediction of off-therapy outcomes [35]. Among 1552 CHB patients, cumulative probability of HBsAg loss was 3.2% at 12 months and 13.0% at 48 months of follow-up. At the end of 48-month follow-up, end-of-treatment serum HBsAg level <1000 IU/mL among Caucasians and serum HBsAg level <100 IU/mL among Asians highly predicted development of HBsAg loss (>30%) [35]. Thus, we may determine serum HBsAg level at the end of NUC treatment for prediction purpose; and off-NUC therapy monitoring is mandatory for all patients receiving NUC therapy.

## 5. Post DAA Therapy for Chronic Hepatitis C in HBV/HCV Co-Infected Patients

In HBV endemic areas, it is not unusual for clinicians to treat patients with concurrent HCV infection [46,47,48]. Concurrent HCV infection has been demonstrated to accelerate the progression of HBV-related liver diseases [46,47,48,49]. HCV has been found to suppress replication of HBV in vitro and clinically in patients with HBV/HCV co-infection. After the clearance of HCV by curative anti-HCV therapy, the suppressive effect of HCV on HBV replication is removed which gives HBV the opportunity to reactivate [36]. Before the introduction of DAA therapy, pegIFN plus RBV was a favored treatment choice for patients with chronic HBV/HCV co-infection; and approximately 60% of the patients were reported to have developed HBV reactivation. Usually, the event occurred either during (~40%) or after (~60%) the treatment course [50].

After DAA was widely used for treating chronic hepatitis C for its efficacy, convenience and safety, HBV reactivation has been documented to be an important issue in co-infected patients [36,37,38,39,40,41,42,51,52]. Chen conducted a systemic review and meta-analysis to investigate and compare the rate of HBVr in CHC patients treated with interferon (IFN)-based therapy and in co-infected patients treated with DAA therapy [39]. In brief, the incidence of HBVr did not differ between the two groups of patients: about 15% for patients receiving IFN-based therapy and 12% for those receiving DAA therapy. One thing worthy noting was that HBVr event occurred earlier in those receiving DAA treatment in comparison with those receiving IFN-based therapy.

The risk of HBVr was also investigated in other clinical studies; and 25% to 87.5% of the HBsAg-positive patients receiving DAAs developed this event [39,41]. These findings suggested that immunologic mechanisms of HBVr should be clarified and prophylactic anti-HBV therapy is essential particularly for cirrhotic patients [40,42].

## 6. Immune Checkpoint Inhibitors for Cancers

Considering the high prevalence of HBV infection in Asia-Pacific region, immune checkpoint inhibitors (ICI) use in patients with HBV chronic viral infection may be common. However, few studies have investigated the risk of HBVr in patients receiving ICI therapy so far, and only few cases of HBVr in patients receiving ICI therapy have been reported in the real world setting [44].

A territory-wide observation study was conducted in Hong Kong recently [45]. In this large cohort study, patients with the prescriptions of ICI from 2014 to 2019 were identified via electronic medical record system, and 990 patients with current HBV infection (HBsAg positive, *n* = 397) or past HBV infection (HBsAg negative but anti-HBs or anti-HBc positive, *n* = 593) were included. All HBsAg-positive patients received oral NUC at the start of ICI therapy while 15.9% of the HBsAg-negative patients also received anti-HBV NUC. The data showed that 39% of the patients with current HBV infection and 30% of the patients with resolved HBV infection experienced AFs (serum ALT level >2 times ULN) of hepatitis B. Reactivation of HBV (≥2 log increase in HBV DNA compared to the baseline level) was found in only two of the HBsAg-positive patients, and none of the patients with resolved HBV infection. These findings strongly supported that the hepatitis flare was common in patients with evidence of HBV infection receiving ICI therapy. However, the majority of the hepatitis flare was not related to HBV reactivation. Overall, the risk of HBVr was very low, particularly among patients receiving concomitant NUC therapy.

The clinical features of the acute flares of chronic hepatitis B in various clinical situations are shown in Table 1.

## 7. Immunopathogenesis of AF of CHB

### 7.1. Potential Role of HBV Genomic Variation

The genome of HBV evolves rapidly during the natural course of chronic HBV infection. Whether such HBV variations could trigger the onset of AF has been investigated prospectively [53]. The chronological full-length viral genomes were sequenced in 14 patients with AF, including 4 patients with spontaneous AF and 10 patients developing AF after receiving medical interventions. The full-length HBV genomes was obtained by polymerase chain reaction and direct sequencing at four time points in individual AF: at baseline, at the peak of serum viral load, at the peak of serum ALT level, and after the peak of serum ALT level. We found that the serum HBV DNA level peaked preceding ALT peak in 13 (93%) of the 14 patients. At the peak of serum HBV DNA level, the serum HBV genome was identical to the one obtained at baseline in 12 patients (86%). After the development of AF, the viral genome evolved in 7 patients (50%) of the viral genomes changed.

Our findings implicated that the majority of AF in patients with chronic HBV infection followed a rapid increase in the replication of a preexisting HBV strain. The development of AF was mainly due to the change of host immune responses instead of the emergence of hepatitis B viral mutations.

### 7.2. Host Immunity and Liver Damage during HBV Infection

HBV-induced liver injury is largely host immune-mediated, because this virus is non-cytopathic [16]. CD8+ T cells are considered the major mediators causing hepatic damage during acute HBV infection, by recognizing and directly attacking the infected hepatocytes [54]. CD8+ T cells produce interferon gamma (IFN-γ) which induces the production of CXCL-9 and CXCL-10, and recruits other inflammatory immune cells, leading to liver damage [16,55].

In contrast, liver damage during chronic HBV infection is mainly attributed to dysregulated activation of intrahepatic immunity leading to nonspecific hepatocyte killing [56,57,58,59]. By acquiring exhaustive phenotypes and producing less inflammatory chemokines and cytokines, it is believed that CD8+ T cells may not be the major mediator of hepatic injury. Instead, the liver injury is driven by other recruited immune cells. Interestingly, associated inflammatory immune markers in CHB patients are fairly similar to those observed in liver damage during acute HBV infection, including type I interferons, chemokines, particularly CXCL-8, CXCL-9 and CXCL-10 [17,60,61,62].

### 7.3. Host Immunity Profile during AF of HBV Infection

Evidence from immunohistologic studies showed that there was CD8+ T cells infiltration and increased production of interferon gamma (IFN-γ) during the acute flare of chronic hepatitis B, as well as increased HBcAg/HBeAg-specific T cells before and during the hepatitis flares [3,4,17]. With high ALT levels, Th1 phenotypic cytokines (IL-2 and IFN-γ) were upregulated, and there was strong correlation among ALT levels, liver injury, and the increased circulating and intrahepatic IL-17 producing CD4+ T cells. In addition to T cells, IL-10-producing regulatory B cells and the serum IL-10 level tended to increase in parallel with the rising serum HBV DNA during the acute flare of hepatitis B, and began to decrease soon after the peak of serum ALT level during the flare [62]. Other cytokines and chemokines were also identified participating in the process of hepatitis flares. Dunn reported that both serum IFN-α and IL-8 levels positively correlated with the increase of serum HBV DNA level right before the elevation of serum ALT; and the rising serum IFN-α and IL-8 would subsequently facilitate natural killer (NK)-cell mediated liver cell damage [61]. High serum IFN-γ inducible chemokines CXCL-9 and CXCL-10 levels were also found to correlate with the development of hepatitis flares [61].

The above findings demonstrated that dynamic interactions of the virus, and innate (including cytokines and chemokines) and adaptive immune responses contribute to the development of hepatitis B flare in patients with CHB [16,57,58,63]. In this review, we will mainly focus on the role of cytokines and chemokines.

## 8. Brief Review of Key Cytokines and Chemokines

### 8.1. TNF-Alpha

In general, TNF-alpha is an inflammatory cytokine produced by macrophages or monocytes during acute inflammation, and in response to infections and cancers [64]. In the scenario of HBV infection, TNF-alpha inhibits HBV replication, provides antiviral immunity, and induces inflammation [3,65]. Serving as a proinflammatory cytokine, TNF-alpha is responsible for a diverse range of intracellular signal pathways. In studies using mice as animal models, TNF-alpha was responsible for viral clearance through modulation of host immune system. In other words, blockage of the function may lead to impaired HBV clearance and an increase of viral load [66].

From another aspect, the production of TNF-alpha is one of the earliest events in many types of liver injury, which triggers the production of other cytokines that may lead to recruitment of other inflammatory cells and the initiation of hepatocyte killing and healing [67]. Studies found that the transcription genes of TNF-alpha and proinflammatory cytokines such as IL-10 are hardly detectable in the normal liver; and the administration of TNF-alpha to animals [68] or the incubation of hepatocytes with TNF-alpha in vitro [67] facilitates hepatocyte proliferation.

Since TNF-alpha is actively involved in systemic inflammation, anti-TNF-alpha has been adopted as a treatment strategy in several inflammatory diseases. We thus have the chance to observe the impact of TNF-alpha inhibition in the development of HBVr. A meta-analysis in 2018 revealed the risk of HBV reactivation in patients with inflammatory arthritis receiving TNF-alpha inhibitors was up to 15.6% [69]. Previous studies also revealed the risk of HBVr in patients with CHB after therapy with TNF-alpha inhibitors such as infliximab, supporting the role of TNF-alpha in viral replication suppression and the warrant of hepatitis B serological marker testing before the initiation of anti-TNF therapy [70,71].

### 8.2. IFN-Gamma

Taking part in both innate and adaptive immunity, IFN-gamma serves as the primary activator of macrophages, and also of neutrophils and NK cells [3,57,58]. The cytokine triggers the differentiation of cytotoxic T cells proliferation, and is able to enhance the microbicidal activity by releasing other cytokines such as TNF-α, IL-1 and IL-6. In non-cytolytic actions, IFN-gamma and TNF-alpha may indirectly enhance the suppression of HBV by mediating the inhibition of HBV gene expression and replication [65]. The cytokine is also found to be associated with antigen-specific tolerance in CHB patients during HBV persistence, where it facilitates the retention of antiviral CD4+ T cells in the liver, possesses antiviral immunity, and further induces inflammation [72]. IFN-gamma had also been found to be involved in the development of hepatitis B flare [73]. Underlying mechanisms and clinical implications of IFN-gamma in the development and therapeutic potential of hepatitis B flare await further studies to clarify.

### 8.3. IL-6

IL-6 is a pleotropic cytokine, serving a pro-inflammatory role in the regulation of the biologic responses of several target cells including hepatocytes [74]. The production of this cytokine is associated with multiple cell types, including fibroblasts, mast cells, macrophages, dendritic cells, T cells and B cells, in response to tissue damage or inflammation, including the scenario of certain viral infections [75,76]. IL-6 attenuates inflammatory responses by suppressing cytokine production and CD4 and CD8 T cells proliferation, and modifies the function of certain antigen-presenting cells [75]. In vitro studies had provided evidence regarding the antiviral effect towards HBV, where the cytokine mediates blockage of HBV infection in hepatocytes by inhibition of the expression of HBV receptors [76,77,78]. As a result, the immune responses of the T cells could be hindered and consequently have a negative impact on the clinical course of chronic HBV infection [79].

The role of IL-6 in the development of hepatitis B flare was investigated previously [74]. Briefly, serum profiles of IL-6 at the onset of acute flare may help identify patients with better clinical outcomes; and targeting IL-6 may be a promising approach in the management of hepatic necroinflammatory activity from the therapeutic view [80].

### 8.4. CCL2

CCL2, known as MCP-1 (monocyte Chemoattractant Protein 1), attracts T cells, dendritic cells and monocytes in patients with active infection, and bridges innate and adaptive immunity [81]. Previous studies suggested that CCL2 may serve as a useful serum marker predicting liver necroinflammatory process in CHB patients, as the serum level of the chemokine in patients with HBV were higher than that in the healthy controls, and also higher in patients with serum ALT level ≥ two times of upper limits of normal than that of patients whose ALT level were <2X ULN [69,82]. Nevertheless, compared the CCL2 level in people with and without HBV infection, and found that the HBV infection group had a lower level of CCL2 [83]. In addition, CCL2 was inversely correlated with HBV viral load and HBsAg level. The role of CCL2 in the natural course and in the outcome prediction of chronic HBV infection is interesting and warrants further investigations.

## 9. Role of Cytokines and Chemokines in AF of Chronic CHB in Various Clinical Situations

### 9.1. Spontaneous Acute Flare of Chronic Hepatitis B

Our previous studies implied that the development of hepatitis B flare was mainly due to the change of host immune responses instead of the emergence of hepatitis B viral mutations [53]. Previous review of individual cytokine or chemokine also suggested several cytokines or chemokines are able to suppress HBV replication or modulate host immune responses. To clarify the role of cytokines and chemokines in the development of hepatitis B flare, we investigated the kinetics of various cytokines/chemokines in patients developing AF of CHB in a prospective manner [74]. We tested the kinetics of 6 serum cytokines and five serum chemokines in 36 HBeAg-positive patients with acute flare of CHB in a prospective way. After a median follow-up of 4 years, we found 22 (61.1%) developed favorable HBeAg seroconversion. The rate of undetectable serum IL-6 level at the onset of hepatitis flare was significantly higher in those who obtained HBeAg seroconversion (86%) as compared to those without HBeAg seroconversion (43%) (*p* = 0.016). The results suggested that serum IL-6 level may be used a serum marker for the prediction of HBeAg seroconversion at the onset of AF.

In another prospective study, we monitored the serum cytokine/chemokine profiles, HBV DNA, and ALT levels in 250 patients [84]. During follow-up, 44 of the 250 patients developed hepatitis B flare. We then analyzed the effect of several factors including the clinical features (age, gender, HBeAg, ALT, HBV genotype), 6 serum cytokines and 5 serum chemokines on the serum HBV DNA dynamics at several time points. We found that the abrupt increase of serum viral load correlated well with the increase of IL-10 and CXCL10/IP-10. Increase of serum viral load was preceded by an increase of serum IL-4, IL-6, and IL-10. Furthermore, we identified that combination of baseline serum IL-6 level, serum ALT level at the peak of serum HBV DNA and HBV genotype reliably predicted the development of subsequent hepatitis B flare. Our data also implied that enhanced Th2 activity is likely involved in the surge of HBV DNA level before hepatitis flare.

Apart from our studies and findings, a prior comprehensive review indicated enhanced expression of Th1 phenotypic cytokines (IL-2 and IFN-γ) [3,25,26], and high serum levels of IFN-γ inducible chemokines CXCL-9 and CXCL-10 [33] during the process of hepatitis flare [26]. Besides, the levels of the programmed cell death protein 1 (PD-1) and its ligand PD-L1 were in parallel with the levels of serum HBV-specific T cells and ALT during the increase, peak and decline phase of hepatitis flare [34]. Overall, the increasing hepatitis activity during AF was found to be correlated well with the up-regulation of IL-2 (major cytokine activating cytotoxic T cell) and IFN-γ. On the other hand, IL-4 was found to have negative regulatory association with the necroinflammatory activity of the liver, and positive association with the replication of HBV in patients with chronic HBV infection [73].

All these data supported that certain cytokines or chemokines, alone or in combination, may help predict the onset and clinical outcomes of patients experiencing AF. Their clinical applications should be investigated and validated in future prospective studies.

### 9.2. Hepatitis B Flare during Treatment of Chronic Hepatitis C by DAA in Patients with HBV/HCV Co-Infection

Direct-acting antiviral agents (DAAs) have become the new standard for the treatment of patients with HCV infection [38,51,52]. However, during the treatment of HCV, HBV may reactivate in patients with HBV and HCV co-infection [36,37,38,39,40,41,42,43]. We thus explored the profiles and predictive value of serum cytokines/chemokines regarding HBV flare [85]. We collected 25 patients who had concurrent HBV/HCV and received DAA therapy for chronic HCV infection. Serial serum cytokine/chemokine levels were tested during the DAA treatment course and thereafter. HBV virologic reactivation was defined by an increase of serum HBV DNA to >10 times and clinical reactivation was defined by >1.5-fold elevation of serum ALT level than nadir and >100 U/L; or >2-fold increase from nadir and greater than the upper normal limit, in addition to virologic reactivation. Finally, 20 (80%) patients developed HBV virologic reactivation and 6 (24%) patients developed clinical reactivation. Pre-treatment serum TNF-alpha was higher, and week 4 serum IFN-gamma level was lower in patients who developed clinical reactivation in comparison to those without clinical reactivation.

Our findings consistently indicated that baseline serum TNF-alpha level and week-4 serum IFN-gamma level served as excellent predictors of HBV clinical reactivation. Prospective studies on large clinical samples will help validate these findings.

### 9.3. Withdrawal Hepatitis Flares Post-NUC Therapy for Patients with CHB

Cytokines and chemokines exhibit both pro-inflammatory and anti-inflammatory activities during the process of AF of CHB [16,64,67]. For example, in patients with spontaneous or NUC withdrawal flare of hepatitis B, increased expression of serum chemokine (C-X-C motif) ligand (CXCL) 9–11, IL-12 and Th1 cytokines had been documented. These data suggested a pro-inflammatory role for these chemokines in the development of hepatitis flare [17,57,86,87]. In patients with NUC withdrawal hepatitis flare, serum IL8 level and serum IFNα level were also found to be increased [17,61], the former promoting TRAIL receptor-2 expression in liver compartment and the latter increasing TRAIL expression in NK cells [61]. These features suggested IL8 and IFNα were involved in NK activation and then contributed to hepatocellular injury. Besides, increased serum CXCL13 and IL21 levels had been demonstrated to correlate with the development of HBsAg seroclearance post NUC withdrawal flare [87].

One recent study from China investigated whether plasma cytokine/chemokine levels could help prediction of HBsAg loss or clinical relapse after stopping NUC treatment [88]. Serial plasma cytokines/chemokines levels were measured for one year post-NUC therapy by commercial kits. They identified that low serum CXCL13 level at the end of treatment predicted the development of CR, and high serum CXCL13 level predicted the development of HBsAg loss. For patients with serum CXCL13 level <80 pg/mL at the end of treatment, the cumulative incidences of clinical relapse was 65% at 4-year follow-up (HR 0.26, *p* < 0.001). For the patients with serum CXCL13 level ≥1000 pg/mL at the end of treatment, 47.5% of the patients developed HBsAg loss (HR 3.01, *p* = 0.008). The authors thus suggested that end-of-treatment CXCL13 level can be an independent predictor of off-treatment clinical outcome.

Previous studies from Taiwan suggested that AFs of CHB are usually self-limiting, accompanied by a decrease in serum HBV DNA level [1,3]. Patients may have the change to obtain HBeAg seroclearance and rarely HBsAg loss (considered as “good” flares). On the other hand, some flares are protracted; thereafter progressive liver injury will be followed. Sometimes severe flares can lead to hepatic decompensation or death (considered “bad” flares) [6,7]. Recognizing and distinguishing “good” and “bad” flares is a challenge clinically; however, clarification of the good versus bad flares is the key to the understanding of natural HBV infection and helps develop therapeutic strategies for the treatment of patients with CHB [64]. Unfortunately, the value of serum cytokine or chemokine profiles in the prediction of good versus bad flares is not investigated yet.

These findings suggest the important role of cytokines and chemokines in the development of AF post-NUC withdrawal. Apart from serum HBsAg level and other serum biomarkers, potential applications of serum cytokines/chemokines and other immunologic markers should be taken into consideration and investigations regarding the development of outcome prediction model.

The findings and implications of various serum cytokines and chemokines in patients with acute flare of chronic hepatitis B in various clinical setting are summarized in Table 2.

## 10. Conclusions

In conclusion, immunopathogenesis of AF is complex and may differ in various clinical situations. Overall, host immune responses play a crucial role in the development of the flare and in the determination of the clinical outcomes. Currently data support that several serum cytokines and chemokines have been found to be involved in the process of acute flares, and may serve as prediction biomarkers of clinical outcomes and even potential therapeutic targets in the future [92]. More prospective studies would be needed to validate these findings.

## Figures and Tables

**Table 1:** Clinical features of acute flare of chronic hepatitis B in various clinical situations.

Clinical Settings	Population at Risk	Features	Reference
Spontaneous AF of Hepatitis B	HBeAg-positive and HBeAg-negative HBsAg carriers	Development phases: HBeAg-positive immune clearance (HBeAg seroconversion) phase and HBeAg-negative reactivation phase HBV reactivation (increase of HBV DNA) and increase of serum HBsAg levels usually occur before the rise of serum ALT level.	[1,2,3,4,5,8,9,10,11,12,13,14]
Cancer Chemotherapy/Immunosuppressive Therapy	Patients with hematologic or other solid cancers Stem cell or solid organ transplantation Trans-arterial chemoembolization of HCC HBsAg-negative anti-HBc positive patients undergoing strong immunosuppressive therapy (such as rituximab)	Serum ALT elevation may occur in-between therapy administrations or after the end of the therapy. Adding corticosteroid is found to increase the risk and severity of HBV reactivation.	[28,29,30,31,32,33]
Withdrawal Hepatitis Flares Post-NUC Therapy	Patients with HBeAg-positive chronic hepatitis B who remain HBeAg positive at the time point of stopping NUC or who receive inadequate consolidation therapy post-HBeAg seroconversion. Patients with HBeAg-negative chronic hepatitis B whose serum HBsAg level is high (for example, >100 ng/mL) at the time point of stopping NUC.	An increase of serum HBV DNA occurs after cessation of NUC therapy, followed by hepatitis flares. The chronological profiles of serum HBV DNA level versus ALT level, and the clinical presentation of the off-therapy hepatitis flares are similar to those occurring during the spontaneous AF of CHB.	[6,7,19,20,21,22,23,24,25]
Post DAA Therapy for Chronic Hepatitis C in HBV/HCV co-Infected Patients	All HBsAg positive and HCV RNA positive patients	HBVr and AF may occur during either during or after the DAA treatment course. HBV reactivation event occurs earlier in those receiving DAA treatment in comparison with those receiving IFN-based therapy.	[37,38,39,40,41,42,43,44,49,50,51,52]
Immune Checkpoint Inhibitors FOR Cancers	Patients with current HBV infection and not receiving NUC prophylaxis.	The risk of HBV reactivation is very low, if HBsAg-positive patients receive concomitant NUC therapy.	[45]

AF: acute flare; HBeAg, hepatitis B e antigen; HBsAg, hepatitis B surface antigen; HBV, hepatitis B virus; ALT, alanine aminotransferase; HCC, hepatocellular carcinoma; NUC: nucleos(t)ide analogue; HCV, hepatitis C virus; DAA, direct acting anti-viral; HBVr, hepatitis B virus reactivation; IFN, interferon.

**Table 2:** Findings and implications of various serum cytokines and chemokines in patients with acute flare of chronic hepatitis B in various clinical setting.

Cytokines or Chemokines	Implications in Patients with AF of CHB	Reference
TNF-alpha	Inhibit HBV replication, enhance anti-HBV immunity and induce inflammation. TNF-alpha was responsible for viral clearance. Risk of HBV reactivation in patients with inflammatory arthritis receiving TNF-alpha inhibitors was up to 15.6%. Decline of serum TNF-alpha level during DAA treatment was associated with HBV clinical reactivation. Administration of TNF-alpha to animals or hepatocytes with TNF-alpha in vitro facilitated hepatocyte proliferation.	[66,67,69,85,89]
IFN-gamma	Suppress HBV replication, anti-HBV immunity and induce inflammation. IFN-gamma was found to be associated with antigen-specific tolerance in CHB patients during HBV persistence, where it facilitated the retention of antiviral CD4+ T cells in the liver, possessed antiviral immunity, and induced inflammation. There was increased production of IFN-gamma during AF. AF in genotype B HBV-infected patients had a significantly higher number of IFN-gamma producing cells (Th1 response) as compared with those in genotype C HBV-infected patients.	[65,67,73]
IL-4	IL-4 was associated with the down-regulation of inflammation in the liver, and was positively correlated with serum HBV DNA levels in CHB.	[73,88]
IL-6	Mediate HBV entry into hepatocytes, inhibit HBV replication and induce inflammation. IL-6 mediated blockage of HBV infection in hepatocytes by inhibition of the expression of HBV receptors, dampened effective T-cell responses, and influenced the clinical course of chronic HBV infection. IL-6 had been demonstrated to suppress HBV replication at the transcription level and correlate with serum ALT level during AF. Frequency of undetectable serum IL-6 level (<3 pg/ mL) at the onset of AF in patients with HBeAg seroconversion was higher in comparison with those with persistent positivity for HBeAg. Combination of HBV genotype, IL-6 at baseline, and ALT level at the peak of serum HBV DNA during AF predicted subsequent development and pattern of hepatitis B flare.	[58,59,74,77,78,79,84]
IL-10	Inhibit cytokine production, regulate T cell immunity and induce immune tolerance to HBV infection Serum IL-10 level increased in parallel with the rising serum HBV DNA level during acute flare of hepatitis B. Serum IL-10 level decreased soon after the peak of serum ALT level during the flare.	[57,63]
CCL2	HBV clinical reactivation was associated with declining on-treatment CCL2, indicating its role against viral replication in host immune system. Decreased level of CCL2 after DAA treatment may contribute to HBV reactivation. CCL2 may serve as a useful serum marker predicting liver necroinflammatory process in CHB patients. CCL2 serum level was higher in patients with HBV, and higher in patients with serum ALT level ≥two times upper limit of normal (2x ULN) than that of patients whose ALT were <2x ULN. There was negative correlation of CCL2 with serum HBV viral load and quantitative serum HBsAg level.	[69,83,90,91]
**Cytokines/Chemokines in Combination**		
TNF-alpha, IFN-gamma, CCL2	Patients with clinical reactivation in HBV/ HCV co-infection had higher pre-DAA-treatment TNF-alpha, lower week-4 IFN-gamma levels, and significant declines of CCL2 and TNF-alpha.	[85]
CXCL10, IL-4, IL-6, IL-10	Upsurge of viral load during hepatitis flare correlated with the increase of IL-10 and CXCL10, and was preceded by an increase in serum IL-4, IL-6, and IL-10.	[84]
IFN-gamma, IL-2	IFN-gamma and IL-2 were upregulated during high ALT levels. Increasing hepatitis activity during AF was found to be correlated with the upregulation of IL-2 and IFN-gamma.	[3,73]
CXCL-9, CXCL-10, IFN-gamma	Hepatitis flare was temporarily associated with high serum levels of CXCL-9 and CXCL-10. IFN-gamma induced the production of CXCL-9 and CXCL-10, and recruited other inflammatory immune cells, leading to liver damage.	[55]
IFN-alpha, IL-8	Serum IFN-alpha and IL-8 levels positively correlated with the increase of serum HBV DNA level right before the elevation of serum ALT. Subsequently, the increased serum IFN-alpha and IL-8 facilitated natural killer (NK)-cell mediated liver cell damage. In patients with NUC withdrawal hepatitis flare, serum IL-8 level and serum IFN-alpha level were found to be increased. IL-8 and IFN-alpha were involved in NK activation and then contributed to hepatocellular injury.	[61,64]
CXCL-13, IL-21	CXCL-13 and IL-21 levels had been demonstrated to correlate with the development of HBsAg seroclearance post NUC withdrawal flare.	[87,88]

AF, acute flare; CHB, chronic hepatitis B; HBV, hepatitis B virus; TNF, tumor necrosis factor; DAA, direct acting antiviral; IFN, interferon; IL, interleukin; HBeAg, hepatitis B e antigen; HBsAg, hepatitis B surface antigen; NUC, nucleos(t)ide analogue.

## Abbreviations

AE, acute exacerbation; AF, acute flare; ALT, alanine aminotransferase; HBeAg, hepatitis B e antigen; HBsAg, hepatitis B surface antigen; HBV, hepatitis B virus; HCC, hepatocellular carcinoma; IL, interleukin.
